# Economic effect of the golf simulation industry in Korea: an analysis based on the SVAR model

**DOI:** 10.1057/s41599-022-01255-9

**Published:** 2022-07-13

**Authors:** Yuanyuan Hao, Mengyuan Kong

**Affiliations:** 1 School of Economics, Jiangsu University of Technology, 213001 Changzhou, China; 2 Department of Physical Education, Dankook University, Yongin-si, 16890 Korea

**Keywords:** Economics, Economics

## Abstract

The main objective of this study is to investigate the impact relationship between the golf simulation industry and economic growth in Korea using impulse response and variance decomposition of Structural Vector Auto-regressive model (SVAR) based on data from 2000 to 2020. The impulse response results show that under the short-term constraint, there is a strong correlation between the golf simulation industry, sports industry investment and labor population, while the interaction between the golf simulation industry, sports industry investment and economic growth is weak, and the trend of their influence is somewhat uncertain under the effect of economic growth. In contrast, there is a unidirectional positive influence effect between the golf simulation industry, sports industry investment, labor population and economic growth under the long-term constraint. However, the difference from short-term constraints is that the long-term shock effect is less volatile with a poorer influence effect, thus leading to the weaker interaction between them. The structural variance decomposition shows that the impact path of the economic effect of the golf simulation industry is the same for both long-term and short-term effects, which indicates that the golf simulation industry, labor population, and sports industry investment all have a positive contribution on economic growth.

## Introduction

Golf simulation refers to a system that can transmit visual, auditory, and tactile stimuli through golf equipment using a screen or loudspeaker to achieve the consistency of virtual experience and authentic experience. Since the system is a virtual golf system based on the screen, it is widely known as “screen golf” in Korea (Lee, 2015). From the operating principle of the golf simulation system, the user can swing the golf club and hit the golf ball on the large screen in front so that the user can play golf on the golf simulation system similar to that on the actual golf course. However, the higher the golf simulation system’s authenticity, the easier it is for the user to get into the competition (game) so that the user can have the virtual experience of playing golf in the virtual interface. In the late 1980s, the golf simulation industry was a sports industry centering in the United States, Germany, Japan, and Korea to develop golf supplies or conduct golf education and training programs. Also, in the golf manufacturing industry, golf club manufacturers have developed the golf simulation system or the golf club swing analyzer to better study and analyze their products and sports track when users use the products. Since 2000, with the rapid development of IT, the golf simulation system has continued to develop and been integrated with other domains, mainly in the form of education, leisure, and entertainment. In recent years, the golf simulation system has been widely used in professional golf teaching systems, characterized by effective and accurate recognition of swing posture. In particular, the golf simulation enjoys relative freedom and diversity without being restricted by space, place, season, and weather conditions. In addition, golf enthusiasts can access golf at a relatively low cost compared with outdoor golf courses. Golf simulation provides learning convenience for beginners of golf and promotes the golf simulation industry to start its business in outdoor golf services in the future. Therefore, golf simulation not only enables users to play remote games on the Internet, but also uses network technology to conduct screen golf league matches. So golf simulation is developing rapidly with a new culture and business model (Ko, 2015).

With the development of the social economy and the change in people’s consumption mindset, indoor golf (golf simulation) has slowly entered the life of the public. Golf is a culture, leisure, and entertainment, a way of life, a fashionable consumption, with its distinct spirit and concept. The shared experience from the simulation sport of golf is not only sports but also the happiness and satisfaction from the inner spiritual life and the health and upward spirit. Golf simulation culture has a shared trait with traditional golf, conveying civilization, health, and gentlemen to the external world. And golf simulation has a low threshold available 365 days a year. It also gives a charm concerning all-day and community-based health, leisure, sports, and civilized social activities. The development of golf simulation can better promote the high-level development of national fitness and better meet the people’s fitness and health needs (Song and Li, 2015). In 2016 when South Korea suffered from economic weakness, the government released a series of initiatives to stimulate the economy, including actively promoting the popularization of golf simulation. The first step to achieving this plan was to actively develop golf simulation courses and simulated golf bars so that many nationals could enjoy the realistic experience of playing golf indoors with merely a tiny amount of money. In addition, in the context of COVID-19, the Korean government’s policy support and the epidemic’s influence have allowed the healthy, green, and civilized golf simulation industry to flourish, promoting Korea as the leading golf simulation sports country in Asia. Furthermore, according to the statistics of the Korean sports industry from 2019, the golf simulation industry had 23,900 employees, accounting for 5.6% of the total number of employees in the sports industry in 2017. There were 466 golf simulation sectors with 4.2879 million users. The total income of the golf industry reached 5.025 billion US dollars, accounting for 9% of the total income of the sports industry. However, the golf simulation industry had 23,200 employees, accounting for 5.3% of the total number of employees in the sports industry in 2018. The number of golf simulation sectors increased to 449, a decrease of 3.65% compared with 2017. The number of users reached 3.4078 million, with a year-on-year decline of 20.50%. The total income of the golf industry reached 5.381 billion US dollars, with a year-on-year increase of 7%, accounting for 11% of the total income of the sports industry. Therefore, the development of the golf simulation industry not only has a particular attraction for domestic golf lovers in Korea but also can increase the corresponding fun and vitality for golf tourists from all over the world, as well as generate a corresponding chain reaction for the development of other related industries (Xu and Yang, 2019). In addition to the coordinated development of the golf simulation industry with existing industries directly related to golf (apparel and golf supplies), the dynamic nature of the golf simulation course can also be adopted to create additional economic added value, such as more jobs and more income for catering. Therefore, Korea has applied virtual and real technologies to business through the development of the golf simulation industry based on the new IT information technologies, and it has been evaluated as the most successful commercial integration model.

In the golf industry chain, although the golf simulation industry has a more significant impact on the economy, there are few research results in this area, especially from an empirical point of view. Thus, it is necessary to study its impact on other industries and economic effects from the perspective of industrial development. Therefore, this study examines the impact of golf simulation on economic growth in Korea from 2000–2020 from the perspective of the sports industry and research results with the employment of the SVAR model to maximize the understanding of the economic effects of golf simulation production, added value, and jobs (labor force) and the feasibility of future industrial development.

The remainder of this paper is structured as follows. Section “Literature review” extensively reviews studies directly or indirectly related to the golf industry and specifies the research gap. Section “Econometric methods and data” introduces the data used in the analysis, presents the estimation method of the SVAR model, and identifies the two shock functions and structural variance decomposition. Section “Results and discussion” breaks down the shocks in the golf simulation industry and economic growth into two types (short-term and long-term) and variance decomposition and presents the empirical analysis results. Lastly, Section “Conclusion and policy recommendations” presents the analysis results’ economic implications, policy recommendations, and limitations.

## Literature review

Foreign research on golf and the golf industry is relatively mature, with most of the literature focusing on research on sporting skills, golf club management and operation, golf tourism, and golf and the environment. For example, Gelan (2003) argues that the organization of golf leagues benefits from the increase in government revenue and the development of urban infrastructure construction. It can also drive the growth of related industries. Poulin et al. (2006) found that the specialization of product production in the golf industry is one of the advantageous factors for golf companies to remain competitive in the fierce competition. Nonetheless, the quality of their products, prices, and timeliness of service have become significant challenges for the survival of golf companies (Zhang et al., 2016). However, Brown et al. (2006) studied the sunk cost, bundling cost, and pricing of golf tourism products by applying the first and third theorems of consumer demand. The study showed that golfers’ travel costs are directly proportional to the golf course, location, and course green costs. This view is supported by the study of Petrick and Backman (2002). Tassiopoulos and Haydam (2008) argue that the golf tourism market can enhance the rich tourism experience and promote the development of sports tourism products and the quality of the surrounding environment (Kim et al., 2005). However, Hodgkison et al. (2007) come to the opposite conclusion and conclude that the vast majority of golf courses are ecologically fragile, lacking in biodiversity, and causing some damage to the environment. On the contrary, Tanner and Gange (2005) and Hwang (2017) supported this view by arguing that the construction of golf courses has contributed to the biodiversity of local birds and insects, as well as the sustainability between the natural environment and golf activities in golf resorts.

However, the development of outdoor golf has been severely constrained by the outbreak of the COVID-19, which is one of the main reasons for the flourishing of golf simulation. The system proposed by Min and Kim (2018) is a virtual golf simulation system with complex playing types. By providing playing modes of hitting actual golf balls and swinging pseudo-golf clubs, players can enjoy various types of golf playing styles according to the user’s preferences. Based on this, Bae et al. (2010) proposed a new sensing method for determining the flight of a golf ball. A new sensing system for extracting features of a golf ball and club was proposed using a perpendicular planar sensor. Furthermore, through a detailed case study, Tadayon et al. (2012) present a design process and framework for SRSG in the context of mixed-reality golf swing simulations. They found that combining swing data with a player’s background (e.g., golf handicap) improved the player’s competitive performance. This is consistent with the study of Kim et al. (2014), who also concludes that the ease of use, performance, fun, and presence of golf simulation all positively contribute to customer productivity and increase users’ willingness. It also has theoretical and managerial implications for increasing customer productivity. Ko (2015) examines the economic effects of golf in Korea using direct and indirect estimation methods. He also finds that golf not only leads to the development of other related industries and economic growth but also increases employment to a certain extent, which is a practical guide to promoting sustainable economic and industrial development.

To sum up, the existing literature mainly examines the impact of outdoor golf on the economy, tourism, and the environment and the effect of golf simulation on players, production and living, and economic development from the perspective of the golf industry. However, there is a lack of systematic and quantitative analysis of studies related to the evolution from the outdoor golf industry to the golf simulation industry (indoor golf). At the same time, since the existing literature analyzes them mainly through questionnaires and data observation, there are too strong hypotheses, thus lacking theoretical and empirical support. Therefore, an analysis of this and the golf simulation shock effect that dynamically ripples to the domestic sports industry market and the macroeconomy is necessary. To gain insight into the economic impacts of the golf simulation industry and to fill the gaps in the existing literature, this study examines the clash between the golf simulation industry and economic growth in Korea from 2000 to 2020 using the SVAR model in a macroeconomic framework, and based on this, proposes corresponding policy recommendations.

## Econometric methods and data

### Unit root test

The purpose of the unit root test is to test the stationarity of series variables, such as GDP per capita (LNPGDP), population of working ages (LNL), sports industry investment (LNSP) and golf simulation industry (LNGF). According to Engle and Granger (1987), if the non-stationary sequence variables can make the original sequence variables stable by linear combination, we can assume that there is a cointegration relationship between the sequence variables. Therefore, we use the Dickey–Fuller (ADF) test (Dickey and Fuller, 1979) to test the sequence and verify whether the sequence is stationary or non-stationary. The least squares (OLS) estimation equation of the ADF test is as follows:

1 $$\Delta X_t = \alpha _1 + \alpha _2T + \delta X_{t - 1} + \mathop {\sum}\limits_{i = 1}^n {\beta _i\Delta X_{t - i} + \varepsilon _i,\quad t = 1,\,2,\, \ldots ,n}$$

where *X*_*t*_ represents any variable (LNPGDP, LNL, LNSP and LNGF) in the model; in the model, assuming that *X*_*t*_ is a non-stationary sequence or *δ* = 0, there must be a unit root among the sequence variables (LNPGDP, LNL, LNSP and LNGF).

### SVAR model settings

The vector autoregressive method (VAR) has the unique advantage of examining endogeneity and multivariate interactions in dealing with time-series data, whereas it is unable to investigate the current period interactions of multiple variables and their intrinsic structure. To improve the VAR by ignoring the shortcoming of present period effects of variables in the VAR, Sims (1986) proposes the structural vector autoregressive model (SVAR). The SVAR model is an extended method based on the VAR model, which introduces various endogenous variables in the economic system into the SVAR model (Dungey and Pagan, 2009; Bala and Alhassan, 2017). It can capture the instantaneous structural relationships between variables within the model system. The issue of too many parameters in the VAR model can be effectively overcome by imposing constraints. Furthermore, it has a more substantial explanatory power for the actual economic performance and is more widely accepted in analyzing the interactions between time series. Therefore, the following SVAR model will be constructed to examine the shock effects using the economic variables of GDP per capita, labor force, sports industry investment, and the golf simulation industry (Adedokun, 2018). The basic equation of the SVAR model is as follows:

2 $$AY_t = {{\Gamma }}_iY_{t - i} + \ldots + {{\Gamma }}_py_{t - p} + u_t$$

$$Y_t = \left( {\begin{array}{*{20}{c}} {y_{1t}} \\ {y_{2t}} \\ {y_{3t}} \\ {y_{4t}} \end{array}} \right),\,A = \left( {\begin{array}{*{20}{c}} 1 \\ { - a_{21}} \\ {\begin{array}{*{20}{c}} { - a_{31}} \\ { - a_{41}} \end{array}} \end{array}} \right.\begin{array}{*{20}{c}} { - a_{12}} \\ 1 \\ {\begin{array}{*{20}{c}} { - a_{32}} \\ { - a_{42}} \end{array}} \end{array}\begin{array}{*{20}{c}} { - a_{13}} \\ { - a_{23}} \\ {\begin{array}{*{20}{c}} 1 \\ { - a_{43}} \end{array}} \end{array}\left. {\begin{array}{*{20}{c}} { - a_{14}} \\ { - a_{24}} \\ {\begin{array}{*{20}{c}} { - a_{34}} \\ 1 \end{array}} \end{array}} \right),\,{{\Gamma }}_i = \left( {\begin{array}{*{20}{c}} {\tau _{11}^i} \\ {\tau _{21}^i} \\ {\begin{array}{*{20}{c}} {\tau _{31}^i} \\ {\tau _{41}^i} \end{array}} \end{array}} \right.\begin{array}{*{20}{c}} {\tau _{12}^i} \\ {\tau _{22}^i} \\ {\begin{array}{*{20}{c}} {\tau _{32}^i} \\ {\tau _{42}^i} \end{array}} \end{array}\begin{array}{*{20}{c}} {\tau _{13}^i} \\ {\tau _{23}^i} \\ {\begin{array}{*{20}{c}} {\tau _{33}^i} \\ {\tau _{34}^i} \end{array}} \end{array}\left. {\begin{array}{*{20}{c}} {\tau _{14}^i} \\ {\tau _{24}^i} \\ {\begin{array}{*{20}{c}} {\tau _{34}^i} \\ {\tau _{44}^i} \end{array}} \end{array}} \right),\,u_t = \left( {\begin{array}{*{20}{c}} {\varepsilon _{1t}} \\ {\varepsilon _{2t}} \\ {\varepsilon _{3t}} \\ {\varepsilon _{4t}} \end{array}} \right)$$

where *Y*_*t*_ is the matrix between endogenous variables of the same period; Γ_*i*_ is the coefficient matrix of lagged *i*th order among the variables; *u*_*t*_ is the vector of random disturbance terms. Therefore, under the condition of the AB-SVAR model, if the influence of exogenous variables is not considered, the SVAR model is constructed for the endogenous variables LNGF, LNPGDP, LNL, and LNSP to examine the economic shock effects as follows (Cassola and Morana, 2004; Ioannidis and Kontonikas, 2008):

3 $$A\left( {\begin{array}{*{20}{c}} {{{LNGF}}} \\ {{{LNPGDP}}} \\ {{{LNL}}} \\ {{ {LNSP}}} \end{array}} \right)_t = {{\Gamma }}_1\left( {\begin{array}{*{20}{c}} {{{LNGF}}} \\ {{ {LNPGDP}}} \\ {{{LNL}}} \\ {{{LNSP}}} \end{array}} \right)_{t - 1} + \ldots + {{\Gamma }}_p\left( {\begin{array}{*{20}{c}} {{{LNGF}}} \\ {{{LNPGDP}}} \\ {{{LNL}}} \\ {{{LNSP}}} \end{array}} \right)_{t - p} + \left( {\begin{array}{*{20}{c}} {u_{{{LNGF}}}} \\ {u_{{{LNPGDP}}}} \\ {u_{{{LNL}}}} \\ {u_{{{LNSP}}}} \end{array}} \right)_t$$

According to Leng (2013), Cholesky decomposition (*Ω* = *P* × *P*′) was conducted on the variance and covariance matrix Ω of the structural impact term *u*_*t*_ of the model 4, as follows:

4 $$A\left( {\begin{array}{*{20}{c}} {u_{{{LNGF}}}} \\ {u_{{{LNPGDP}}}} \\ {u_{{ {LNL}}}} \\ {u_{{{LNSP}}}} \end{array}} \right) = B\left( {\begin{array}{*{20}{c}} {\varepsilon _{{ {LNGF}}}} \\ {\varepsilon _{{{LNPGDP}}}} \\ {\varepsilon _{{{LNL}}}} \\ {\varepsilon _{{ {LNSP}}}} \end{array}} \right),\,{\rm{where}}\left( {\begin{array}{*{20}{c}} {\varepsilon _{{ {LNGF}}}} \\ {\varepsilon _{{{LNPGDP}}}} \\ {\varepsilon _{{{LNL}}}} \\ {\varepsilon _{{{LNSP}}}} \end{array}} \right) = p^{ - 1}\left( {\begin{array}{*{20}{c}} {u_{{{LNGF}}}} \\ {u_{{{LNPGDP}}}} \\ {u_{{{LNL}}}} \\ {u_{{ {LNSP}}}} \end{array}} \right)$$

Since the economic effect of the golf simulation industry has four endogenous variables, it is necessary to impose four constraints on the effect impact model. Therefore, according to the different methods of imposing constraints on the SVAR model, it can be divided into short-term and long-term constraints (Cassola and Morana, 2004; Ioannidis and Kontonikas, 2008; Dungey and Pagan, 2009). The first constraint on the model is a short-term constraint. The short-term constraints are imposed on the research model according to the relevant economic theories. Firstly, suppose the golf simulation industry (LNGF) in the current period does not change with the changes in GDP per capita (LNPGDP), the population of working ages (LNL), and sports industry investment (LNSP) in the current period. In that case, it means that the golf simulation industry may be more influenced by the economic effect of the previous period and the expected future economic shock effect. Therefore, in the influencing factors in constraint A that _12_= _13_= _14_ = 0. Secondly, the GDP per capita does not change with the labor population, and sports industry investment implies some lagged effect of labor population and sports industry investment in the current period, i.e., _23_=_24_ = 0. Finally, the fact that the labor population also does not change with the sports industry investment in the current period implies that there is also a specific lagged effect of sports industry investment, i.e., _34_ = 0. Model 4 can be sorted out as follows:

5 $$\left( {\begin{array}{*{20}{c}} {\begin{array}{*{20}{c}} {\begin{array}{*{20}{c}} 1 & 0 & 0 \\ 0 & 1 & 0 \\ 0 & 0 & 1 \end{array}} \\ {\begin{array}{*{20}{c}} {a_{41}} & {a_{42}} & {a_{43}} \end{array}} \end{array}} & {\begin{array}{*{20}{c}} {\begin{array}{*{20}{c}} 0 \\ 0 \\ 0 \end{array}} \\ 1 \end{array}} \end{array}} \right)\left( {\begin{array}{*{20}{c}} {u_{{ {LNGF}}}} \\ {u_{{{LNPGDP}}}} \\ {u_{{{LNL}}}} \\ {u_{{{LNSP}}}} \end{array}} \right)_t = \left( {\begin{array}{*{20}{c}} {\begin{array}{*{20}{c}} {\begin{array}{*{20}{c}} {b_{11}} & 0 & 0 & 0\\ 0 & {b_{22}} & 0 & 0\\ 0 & 0 & {b_{33}} & 0\\ 0 & 0 & 0 & {b_{44}} \end{array}} \end{array}} \end{array}} \right)\left( {\begin{array}{*{20}{c}} {\varepsilon _{{{LNGF}}}} \\ {\varepsilon _{{{LNPGDP}}}} \\ {\varepsilon _{{{LNL}}}} \\ {\varepsilon _{{{LNSP}}}} \end{array}} \right)_t$$

The second constraint on the model is the long-term constraint. According to the property setting of the cumulative long-term impulse response of structural disturbance terms proposed by Blanchard and Quah (1988), the fundamental reason is to define the relationship between matrices A and B under short-term conditions and matrix C under long-term conditions (Neusser and Kugler, 1998). Assuming that the influencing factor of endogenous variables *c*_*i*,*j*_ = 0 and *i* ≠ *j*, the model of long-term constraint is adjusted as follows:

6 $$\left( {\begin{array}{*{20}{c}} {{{LNGF}}} \\ {{{LNPGDP}}} \\ {{{LNL}}} \\ {{{LNSP}}} \end{array}} \right)_t = \left( {\begin{array}{*{20}{c}} {\begin{array}{*{20}{c}} {\begin{array}{*{20}{c}} {c_{11}} & {c_{12}} & {c_{13}}&{c_{14}} \\ 0 & {c_{22}} & {c_{23}}&{c_{24}} \\ 0 & 0 & {c_{33}}&{c_{34}}\\0 & 0 & 0 & {c_{44}}\end{array}} \end{array}} \end{array}} \right)\left( {\begin{array}{*{20}{c}} {\varepsilon _{{{LNGF}}}} \\ {\varepsilon _{{{LNPGDP}}}} \\ {\varepsilon _{{ {LNL}}}} \\ {\varepsilon _{{{LNSP}}}} \end{array}} \right)_t$$

### Data selection

The purpose of this paper is to study the economic effect of the golf simulation industry based on the Structural Vector Auto-regressive model (SVAR) and put forward reasonable suggestions for the development of the industry. Since most of the relevant research is mainly theoretical, there are few research results on the economic effect of the golf simulation industry (Watson et al., 2008). Therefore, based on the annual data of Korea from 2000 to 2020 (data sources: Korea Sports Industry Report, Korea National Statistical Office, and World Bank), this paper examines the linkage mechanism between the development of the golf simulation industry in Korea and economic growth. Aside from the total business revenue and economic growth variable (GDP per capita) of the golf simulation industry adopted in this paper, the labor force (economically active population) variable and the sports industry investment variable can reflect the macroeconomic dynamics of Korea selected as follows.

1. *Economic growth (GDP)*: GDP per capita measures Korea’s economic growth to eliminate the price factor. Also, GDP per capita is an effective tool for nationals to understand and grasp the macroeconomic performance of a country or region as an essential indicator of the economic development of a country or region.
2. *Golf simulation industry (GF)*: The total business income of the golf simulation industry is used to measure the industry’s impact on economic growth among all industries. It can also accurately measure the development of the golf simulation industry.
3. *Labor force (L)*: Using the economically active population to examine the contribution of the Korean labor force to industry and economic development, it is also possible to accurately grasp the current status of the labor force providing various economic production and service activities over a certain period. It is also an important indicator to measure a country or region’s labor force participation rate and employment rate.
4. *Sports industry investment (SP)*: The sports industry investment index is used to examine the trend and extent of capital investment in Korea’s sports industry and the change in earnings of the project over a certain period.

Furthermore, considering the general principles of comprehensiveness and availability of data, all variables in the specified model are processed logarithmically. Such interconversion can help obtain a better normal distribution of all data and improve the critical problem of heteroskedasticity, making the final result meaningful and easy to interpret. The results of descriptive statistics in Table 1 show that the interquartile range (IQR) of all variables has no outliers.

**Table 1:** Descriptive statistics of variables used.

Economic variables	LNGF	LNGDP	LNL	LNSP
Mean	3.558	10.087	8.316	4.495
Median	3.424	10.104	8.339	4.496
Max	4.013	10.270	8.401	4.757
Min.	3.023	9.857	8.012	4.199
Std. dev.	0.334	0.128	0.097	0.165
Skewness	0.015	−0.272	−2.159	−0.176
Kurtosis	1.549	1.945	7.579	2.128
IOR	0.548	0.2001	0.081	0.241

## Results and discussion

### Unit root test

According to the results of the unit root test in Table 2, all variables in the model, GDP per capita (LNPGDP), population of working ages (LNL), sports industry investment (LNSP) and golf simulation industry (LNGF), are unstable time series at their original level. However, after the first-order difference, they tend to be stable. Therefore, we can conclude that all sequence variables are the first-order integral I or I(1)-order integral at the significance level of 5% (Chontanawat, 2020).

**Table 2:** Unit root test results.

Sequence	ADF		DF-GLS		PP	
	Level	First difference	Level	First difference	Level	First difference
LNGF	−4.058	−5.369***	−2.754	−5.433***	−4.058	−5.435***
LNPGDP	−3.120	−3.743**	−2.754	−3.711***	−4.058	−4.569***
LNL	−4.058	−5.740***	−2.754	−5.995***	−4.058	−12.34***
LNSP	−4.122	−4.269***	−2.772	−4.482***	−4.058	−6.660***

**, *** Denote rejection of null hypothesis at 5%, 1% significance.

### Test of the SVAR model

**Fig. 1:**
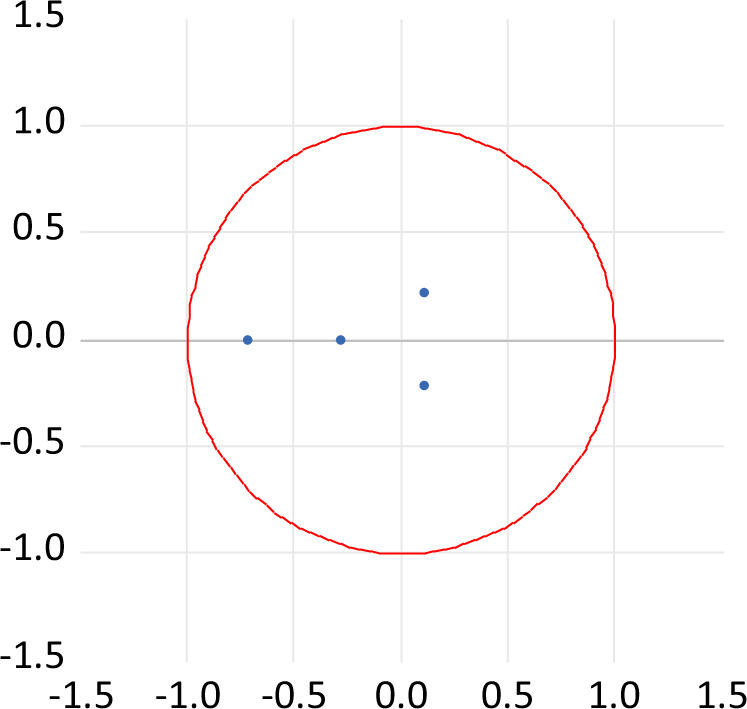
Inverse roots of AR characteristic polynomial.

The lag order in the SVAR model is determined based on the test of the lag order of the VAR model, the optimal lag order (see Table 3), and the stability of the model (see Fig. 1) of the economic effect model of the composite golf simulation industry are determined by using the information criteria of LR, FPE, AIC, HQIC, and SBIC (Cassola and Morana, 2004; Ioannidis and Kontonikas, 2008). Firstly, according to Table 2, the optimal lag order of the golf simulation industry model is 1, and the AR root of each endogenous variable under the optimal lag period is in the unit circle, indicating that the SVAR model of the economic effect of the golf simulation industry is stable. However, for the long-term and short-term structural constraints imposed in the SVAR model on the economic effect of the golf simulation industry, the Johansen cointegration test was performed on the model under the condition of the VAR model (see Table 4). Secondly, according to the test results in Table 4, both the cointegration trace test and the maximum eigenvalue test of the model reject the hypothesis that there is at most one cointegration relationship at the significance level of 5%. Therefore, there are two cointegration relationships (long-term equilibrium relationship) in the SVAR model of the economic effect of the golf simulation industry (Bala and Alhassan, 2017; Chontanawat, 2020). Finally, we need to test the stability of the whole system based on the SVAR model to determine the smoothness of the model. If the estimated AR roots’ inverse falls within the unit circle, then the model is stable. In the case of the golf simulation industry economic effects model, all of its characteristic roots are within the unit circle (see Fig. 1), which means that the model satisfies the smoothness condition and ensures that the impulse response and variance decomposition are performed efficiently.

**Table 3:** SVAR lag order selection criteria.

Lag	LogL	LR	FPE	AIC	SIC	HQIC
0	61.539	NA	1.68e−09	−8.852	−8.678	−8.887
1	106.651	55.562*	5.64e−11*	−12.379*	−11.465*	−12.463*
2	153.024	29.642	6.42e−13	−18.004	−16.439	−18.325

*LR* sequential modified *LR* test statistic; *FPE* final prediction error; *AIC* Akaike information criterion; *SIC* Schwarz information criterion; *HQIC* Hannan–Quinn information criterion.

* Indicates lag order selected by the criterion (significant at 5% level).

**Table 4:** Results of Johansen co-integration test.

Hypothesized no. of cointegrating vectors	Eigenvalue	Trace statistic	Max-eigen statistic
None*	0.998	119.095	86.989
At most 1*	0.841	32.106	23.873
At most 2	0.308	8.233	4.783
At most 3	0.233	3.450	3.450

*Denote rejection of null hypothesis at 5% significance.

### Impulse response analysis

When analyzing the SVAR model, one should focus on how to use the impulse response function (IRF) to portray the dynamic effects on the system when an error term changes or when the model is subjected to some impact. Therefore, this paper analyzes the systematic impact of economic effect perturbation term shocks and the dynamic response of each perturbation term to each other shocks in the golf simulation industry by drawing out impulse response plots.

#### Structural impulse response under short-term constraints

**Fig. 2:**
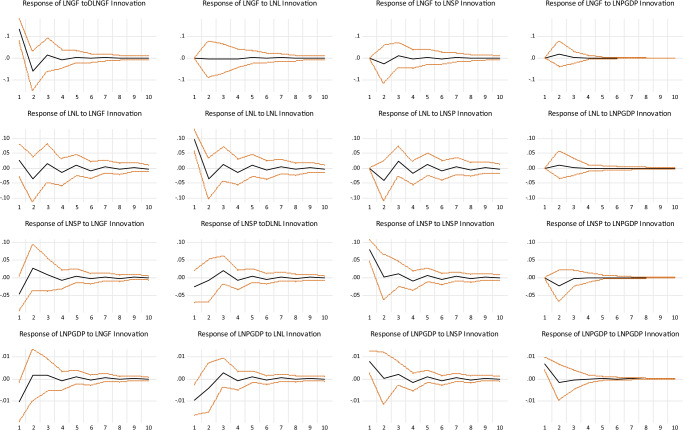
The results of impulse response analysis of four variables to the short-term structural shocks. Estimates with one standard error band and the red line represents confidence intervals were constructed using a recursive−design wild bootstrap, and the black line represents the median.

Under the short-term constraint, we use 10 periods as the lag interval of the impulse response and examine the shock effects of the golf simulation industry, labor force, sports industry investment and economic growth variables, respectively (see Fig. 2). From the overall response curves, we can easily see that all response curves converge to zero over time, which indicates that the SVAR model is smooth under the short-term constraint.

Firstly, the impulse response of LNGF under the Cholesky one standard deviation is shown that when the golf simulation industry is subject to a positive shock in the current period, the labor force and economic growth, and the golf simulation industry itself all have a positive effect on it, and followed by a brief negative effect. However, the sports industry investment has a temporary negative effect on the golf simulation industry that first falls and then rises, which may be the result of the expansion mechanism of the golf simulation industry that makes the share in the sports industry market increase, leading to the irrational development of regional related industries. Overall, the shock effect on the golf simulation industry is 0.002, and the positive and negative impacts keep going back and forth in the interval of [−0.06, 0.13]. The shock effect keeps weakening with time, and the positive effect is greater than the adverse effect. The reason for the above phenomenon is that the market concentration of the golf simulation industry is in a “U-shaped” development process. During its expansion phase, most of the investors in the sports industry have gained greater market power mainly through the individual expansion of business scale. Therefore, the market concentration level of the golf simulation industry is in a decentralized competitive market structure, but the strength of monopolistic competition has noticeable regional differences. In addition, the high concentration of labor force and related sports industry investments in developed regions is the main reason for the regional differences in the golf simulation industry.

Second, the impulse response of LNL under the Cholesky one standard deviation is shown that when the labor force is subjected to a positive shock in the current period, economic growth and the golf simulation industry, and the labor force itself all have a positive effect on it, followed by a short-lived negative effect. However, the sports industry investment has a negative shock effect followed by a short-lived positive effect. In terms of the shock effect on economic growth, the golf simulation industry and sports industry investment significantly impact the change in the labor force, but the result is unstable with fluctuations. This implies that investment in the golf simulation and sports industry can provide more jobs in the short term, which positively contributes to increasing workers’ income and consumption level. Overall, the shock effect on the labor force is 0.001, which is positive and negative in the interval of [−0.037, 0.096], and the shock effect decreases over time. The fluctuation trend of the golf simulation industry and sports industry investment is highly correlated with the fluctuation trend of labor population, which implies a strong correlation between the golf simulation industry, sports industry investment, and labor population in the short term. To a certain extent, the golf simulation industry’s development depends on the region’s labor population and sports industry investment capital.

Third, the impulse response of LNSP under the Cholesky one standard deviation is shown that when the sports industry investment is subjected to a positive shock in the current period, the golf simulation industry and labor population, and economic growth all have a negative effect on sports industry investment, and followed by a brief positive effect. However, sports industry investment has an “L” shaped positive effect on itself, while economic growth has a less volatile effect on it, and the result tends to be stable. This indicates that the impact of economic growth on sports industry investment in the short term is small, and it is challenging to achieve immediate results; that is, the relationship between them is relatively weak. Overall, the average shock effect on sports industry investment is 0.0007, and the positive and negative impacts are repeated in the interval [−0.046, 0.028] and weaken over time. The reason for the above phenomenon is that, with the increase of population aging, the age growth of the working population gradually shows a “U-shaped” trend, which directly leads to the development of sports and leisure-related industries in the short term with a lag, moving the working population in the sports industry increase, which leads to the decrease of the contribution of the sports industry to the national economic growth.

Finally, the impulse response of LNPGDP under the Cholesky one standard deviation is shown that when the economic growth is subject to a positive impact in the current period, the impact of economic growth itself and the impact of investment in the sports industry both have an “L” shaped positive shock effect on it. However, the golf simulation industry and the labor force have a negative shock effect on it, and the response to this impact is weakening as time goes on. While compared to the effects of economic growth itself, the reaction of the golf simulation industry is lagging. However, the shock effects of the golf simulation industry, labor force, and sports industry investment are persistent and more volatile than the effect of economic growth itself. This suggests a long-term solid correlation between the golf simulation industry labor force, sports industry investment, and economic development. The shock effect on the golf simulation industry and labor population is an impact process from negative to a positive outcome. This negative effect gradually disappears and shifts to a positive effect over time. The reason for the above phenomenon is that, with the development of a high-quality economy, the age group of the labor force shows a “U-shaped” trend, which cannot stimulate the growth of the golf simulation industry and traditional sports industry in the short term, thus leading to the negative to the positive impact of golf simulation industry and labor force on the development of the real economy. Besides, there is significant uncertainty in the interaction effect of the golf simulation industry and labor population, depending on the situation.

#### Structural impulse response under long-term constraints

**Fig. 3:**
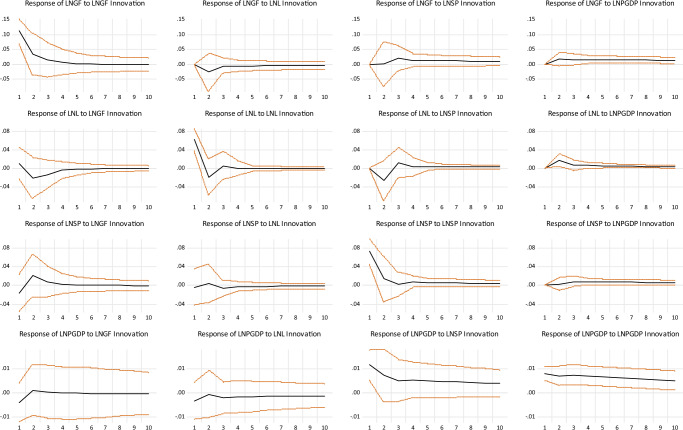
The results of impulse response analysis of four variables to the long-term structural shocks. Estimates with one standard error band and the red line represents confidence intervals were constructed using a recursive-design wild bootstrap, and the black line represents the median.

According to the long-term constraint imposed on the economic effect model of the golf simulation industry, the IRFs among the variables can be obtained (see Fig. 3). First, following a positive shock, the golf simulation industry will have a positive shock effect on itself, which will first decline, then rise and gradually weaken. The impact on the labor force is highly consistent with the path of the short-term constraint, while unlike the short-term constraint, both sports industry investment and economic growth have a persistent positive impact on it. Thus, in the long term, there appears to be a close relationship between the variables.

Second, suppose a long-term constraint is imposed on the labor force. In that case, it is subject to a positive shock that produces an impact process from positive to adverse shock effects on itself, eventually converging to 0. Unlike the short-term constraint, economic growth has a continuous positive shock effect on it, and the long-term shock effect on the labor force is relatively short-lived. In the long term, as the economy grows, the demand for the labor force in the relevant industries will increase.

Third, if a long-term constraint is imposed on sports industry investment, it will be subject to a persistent positive shock effect on itself and economic growth. At the same time, the shock effect on the labor population is mainly consistent with the short-term constraint. In the long term, economic growth can promote the scale of the sports industry. But due to the sports industry being highly concentrated in the service sector, the mobility of the labor force is considerable, i.e., there is a weak correlation between the labor force and the sports industry.

Finally, suppose a long-term constraint is imposed on economic growth. In that case, economic growth will be subject to persistent positive shock effects from both the sports industry and itself, but the shock effects all trend downward. Similar to the short-term constraint, the labor force has essentially the same effect on itself, the golf simulation industry, and economic growth in the long term. Economic growth brings all sustainable positive shock effects, which is a strong validation of the importance of encouraging economic development to some extent. Overall, economic growth, the golf simulation industry, and sports industry investment all focus on positive effects on themselves. In contrast, economic growth has obvious externalities, effectively driving the development of related industries while promoting their development, but there is uncertainty about its impact on the working population. In other words, it still takes a long process to realize the interaction between the development of the golf simulation industry and economic growth.

### Variance decomposition

Variance decomposition is the process of analyzing the extent to which each structural shock in the SVAR model contributes to the variance of the prediction errors of the study variables, so as to further evaluate the relative importance of changes in specific variables to each structural shock in order to better uncover the sources of endogenous variable changes. Variance decomposition, thus, can describe the relative importance of different impacts to the golf simulation industry, labor force, sports industry investment, and economic growth (see Table 5) (Zeng et al., 2020). Firstly, the results of the variance decomposition of the golf simulation industry suggest that in the early years the golf simulation industry was subjected to volatile impacts mainly from its own influence. Although the impact gradually decreases over time, it still remains at a high level of 93.95%. However, the impact of labor force and economic growth on the golf simulation industry is negligible, while the impact of sports industry investment is slightly larger than the impact of labor force and economic growth, and its shock effect is stable at 4.63%. Secondly, the results of the variance decomposition of the labor force show that the labor force is mainly affected by its own fluctuations, but the impact of the golf simulation industry on it is also very significant. Over time, the impact of the labor force itself gradually decreases, and its shock effect stabilizes at 47.5%. The impact of golf simulation industry and sports industry investment gradually increases, and their shock effects stabilize at 15.95% and 10.26%, respectively, which indicates that golf simulation industry and other sports industries still play an important role in the industrial structure of Korea. Third, the results of variance decomposition of sports industry investment show that the impact of impacts on sports industry investment mainly comes from the golf simulation industry, economic growth and sports industry investment itself, and their shock effects are stable at 26.55%, 33.58%, and and 28.57%, respectively. However, the impact of the labor force is relatively small, rising from 5.24% in period 1 to 11.3% in the stabilization period, which indicates that the impact of economic growth on the level of investment in the sports industry is minimal. Therefore, the development of related industries and the working population are the most critical factors in measuring economic development in an open economy. Finally, the variance decomposition of economic growth results shows that the impact on economic growth comes mainly from itself and the golf simulation industry, whose effects are stable at 57.5% and 32.68%, respectively. Compared with the impact of the golf simulation industry and economic growth itself, although the impact on the labor population and the impact of the sports industry is relatively more minor, they are still stable at 7.81% and 2%, which indicates that the golf simulation industry, labor population and sports industry investment all have positive effects on economic growth.

**Table 5:** Results of variance decomposition of LNGF, LNGDP, LNL, and LNSP.

Period	Variance decomposition of LNGF				Variance decomposition of LNGDP			
	LNGF	LNGDP	LNL	LNSP	LNGF	LNGDP	LNL	LNSP
1	100.0	0.000	0.000	0.000	35.158	64.842	0.000	0.000
2	94.92	0.044	0.551	4.483	33.632	61.693	4.137	0.538
3	94.32	0.464	0.613	4.606	32.917	59.188	6.388	1.507
4	94.15	0.464	0.741	4.641	32.767	58.401	7.074	1.759
5	94.05	0.463	0.817	4.670	32.716	57.971	7.442	1.870
6	94.00	0.463	0.855	4.681	32.698	57.733	7.630	1.939
7	93.97	0.463	0.876	4.687	32.689	57.613	7.724	1.974
8	93.96	0.463	0.886	4.690	32.685	57.552	7.772	1.991
9	93.95	0.463	0.892	4.691	32.683	57.520	7.796	2.000
10	93.95	0.463	0.895	4.692	32.682	57.504	7.809	2.005
**Period**	**Variance decomposition of LNL**				**Variance decomposition of LNSP**			
	**LNGF**	**LNGDP**	**LNL**	**LNSP**	**LNGF**	**LNGDP**	**LNL**	**LNSP**
1	6.8489	43.669	49.482	0.0000	23.142	40.783	5.2389	30.835
2	14.054	30.527	46.431	8.9885	27.666	35.734	6.7932	29.807
3	14.830	28.603	46.978	9.5890	26.663	34.765	9.7096	28.862
4	15.376	27.437	47.298	9.8890	26.557	34.167	10.515	28.762
5	15.670	26.848	47.398	10.084	26.538	33.891	10.905	28.666
6	15.810	26.564	47.453	10.173	26.543	33.732	11.108	28.617
7	15.882	26.420	47.480	10.218	26.548	33.650	11.209	28.593
8	15.919	26.346	47.494	10.241	26.551	33.609	11.260	28.580
9	15.937	26.308	47.502	10.253	26.552	33.587	11.287	28.574
10	15.947	26.289	47.505	10.259	26.553	33.576	11.300	28.570

Cholesky ordering: LNGF, LNGDP, LNL and LNSP.

## Discussions

According to the existing research related to the golf industry, most explore the future direction of golf development from the aspects of theory, sports skills, golf course management, and operation. Still, there is a lack of systematic research on the economic effects of golf in the framework of the development of outdoor golf in the golf simulation industry (Petrick and Backman, 2002; Gelan, 2003; Poulin et al., 2006; Hodgkison et al., 2007; Ko, 2015; Hwang, 2017). Therefore, we take the relevant study by Adedowan (2018) as the baseline model and extend the economic effects model as well as analyze the impact of golf simulation industry impacts on economic growth by constructing a special model.

Our first finding supports the existence of a long-term shock effect between the golf simulation industry, economic growth, sports industry investment, and the labor force since any pair of these three variables are bidirectional. In addition to contributing to the extensive literature on outdoor golf and economic growth, the environment, and tourism, our study builds on previous research and expands upon it. Specifically, some studies have only confirmed the effects of golf industry development on economic growth, environment, and tourism (Kim et al., 2005; Ko, 2015; Hwang, 2017) yet ignoring the economic effects of golf industry restructuring, which are a more significant influential factor, especially in the context of COVID-19.

Another significant contribution of our study to the existing literature is related to the consideration of the golf industry. Firstly, it is not difficult to find that economic growth, the golf simulation industry, and sports industry investments all focus on positive effects on themselves. Since all of the effects of economic growth are sustainable and have positive shock effects, this is a strong validation of the importance of encouraging economic development to a certain extent. However, economic growth has an apparent externality that effectively drives the development of related industries and the increased demand for labor in the labor market while promoting its development (He, 2018). Secondly, with the high-quality development of the economy, the age group of the Korean workforce shows a “U-shaped” trend, which will directly lead to a severe lag in the development of sports and leisure-related industries, especially in the context of COVID-19 outdoor golf industry development is severely constrained, which also prompted the restructuring of the golf industry (Zhang et al., 2016). In the context of the uncontrollable environment, the open and tolerant Korean golf practitioners seized the opportunity to explore innovation, broke the traditional business model, and transformed the indoor innovative golf model (golf simulation) to push the golf game to a brand new development process. Finally, we also find that the market concentration of the golf simulation industry is in a “U-shaped” development process, which indicates that the market concentration level of the Korean golf simulation industry is in a decentralized competitive market structure. As we predicate, regional differences in golf simulation are directly or indirectly caused by the high concentration of labor force and related sports industry investments in developed regions (Xu and Yang, 2019; Yang et al., 2020).

In the context of the global COVID-19, people pay more attention to physical health and admire outdoor sports, which has led to a blowout in the development of golf simulation. Regarding breadth, golf simulation has dramatically lowered the threshold for new learners with its unparalleled advantages of a warm winter and cool summer learning environment, rich and exciting teaching methods, and lack of time constraints. It also allows the public to feel the charm of golf. From a deep perception, from the perspective of the long-term growth of junior players, golf simulation can also contribute to a closer link between golf and the future development of junior players with a unique long-term education program and innovative training model and achieve the accelerated growth of the competitive level of golf in all countries. Therefore, under the premise of not violating national policies and regulations, governments worldwide should actively promote the development of golf.

## Conclusion and policy recommendations

### Conclusion

This paper set the stability indicators of economic effects using principal component analysis, and we constructed Structural Vector Auto-regressive (SVAR) model for the stability variables of golf simulation industry, labor population, sports industry investment and economic growth based on the data related to the economic effects of golf simulation industry in Korea from 2000 to 2020. Firstly, with regard to the analysis, we perform auto-regressive distributional lags and structural auto-regressive tests on the SVAR model, respectively, to understand the extent to which economic growth is affected by golf simulation industry impacts. On the one hand, prior to conducting the analysis, we also performed unit root tests on all variables and determined that all variables were first-order single integer I or I(1) order integral series at the 5% significance level. On the other hand, the SVAR model test results indicate that the optimal lag order of the golf simulation industry economic effects model is 1 and that there are two co-integrating relationships (long-term relationships) at the 5% significance level. Furthermore, the statistical robustness test shows the stability of the model and the absence of serial correlation. Secondly, the interrelationships between the golf simulation industry, labor force, sports industry investment, and economic growth in Korea were investigated using impulse response and variance decomposition. The impulse response analysis results show a strong correlation between the golf simulation industry, sports industry investment, and labor population under the short-term constraint. In contrast, the interaction between the golf simulation, sports industry investment, and economic growth is weak. Its impact trend is somewhat uncertain under economic growth, but its shock effect is mainly dominated by a positive response impact in the short term. With the development of a high-quality economy, the age group of the Korean workforce is showing a “U-shaped” trend, which will not stimulate the growth of the golf simulation industry and the traditional sports industry shortly. In addition, the high concentration of labor force and related sports industry investment in developed regions will lead to profound regional differences and a fragmented competitive market structure in the development of the golf simulation industry. The long-term constraint results show that economic growth, the golf simulation industry, and sports industry investments all focus on generating a positive response to themselves. As economic growth has apparent externality, it can effectively drive the development of related industries while promoting its development, which to a certain extent better verifies the importance and objective inevitability of encouraging and supporting the development of small, medium, and micro-enterprises. Different from the short-term constraint, the long-term shock effect between the labor force and sports industry investment is relatively stable, indicating that their interaction is weak; but this effect is relatively long-lasting (Tassiopoulos and Haydam, 2008; Ko, 2015; Zafar, 2017; Xu and Yang, 2019). Overall, the impact paths of the economic effects of the golf simulation industry are primarily consistent for both short-term and long-term effects, with positive effects of the golf simulation industry, labor force, and sports industry investment on economic growth (Zhang et al., 2016; Xu and Yang, 2019; Yang et al., 2020). Finally, the structural variance decomposition results are consistent with the results of the impulse response analysis based on the SVAR model. With the development of the economy, golf simulation has flourished, making this noble sport, which originated in Scotland, a popular recreational activity for all people in Korea. However, in the context of COVID-19, as the development of outdoor golf is severely constrained, the Korea Golf Association has taken “golf for the masses” as a breakthrough. It not only brings into play the traditional advantages of outdoor golf with open space and non-physical contact but also achieves innovative development, creates a healthy lifestyle, promotes the deep integration of golf simulation with economy and health, and strives to achieve a healthy, green and civilized growth of the golf simulation industry.

### Policy recommendations

1. The development of the golf simulation industry requires a strategy. The future development of golf needs not only on-course customers but also needs to convert the general population into potential customers. Therefore, to address the topics faced by indoor golf courses and conceive new growth strategies, marketing strategies that target only on-course golf customers encounter limitations. In addition, the golf simulation industry development generally targets only the customers of outdoor golf courses, and the customers are also mainly high-end groups, so this marketing strategy has reached the limit of industry development to solve the problems faced by golf courses and to develop a new long-term growth strategy. Therefore, in the future development of the golf simulation industry, the marketing strategy must be changed from the traditional golf course business strategy while making the golf simulation a game place. Developing new customers from various angles and a new growth strategy is necessary.
2. The transformation of customer base. Traditional golf customers must shift from the high-end customer base to the middle and low-end customer base but also need to give priority to the residents of the area where the golf course is located while providing specific policies. In other words, through the golf simulation in the community, training colleges, KTV and game halls, etc., it is required to maximize the use of indoor golf simulation for outdoor golf to cultivate low-end and middle-end customer base and use the business resources of indoor golf simulation to create economic benefits and promote the development of household golf courses. Therefore, this strategy not only provides a platform to showcase golf simulation, but also allows more middle- and lower-income groups to understand golf simulation and experience the fun of golf simulation, and increases the visibility and intimacy of golf courses and the game of golf, thus increasing the possibility of establishing new markets.
3. The diversification of business strategies in the golf simulation industry. In other words, efforts shall be made to create a golf tourism industry chain that combines golf simulation with outdoor golf. In addition, golf simulation can enter not only hotels to provide basic entertainment for tourists but also parks, amusement parks, shopping malls, and hospitals to make full use of the practicality and economic benefits of the golf simulation industry to promote the comprehensive development of the industry. In conclusion, the business and survival strategy of golf simulation courses can be said to expand the market as they build networks with the external environment. As a result, such an industrial strategy will undoubtedly become the mainstream industry in the future development of the sports industry, being the inevitable trend of the golf industry restructuring and supply-side structural reform.

In this paper, the research on the economic effect of the golf simulation industry has some limitations. Still, in the future economic development, the golf simulation industry is expected to positively impact the growth of leisure sports, the change of game culture, and other aspects of social and cultural life. Therefore, in this paper, the economic effect of the golf simulation industry is analyzed through statistical methods and econometric models, which lays a foundation for establishing policy support, and is expected to become the basis for analyzing the economic effect of other simulated sports industries in the future.

## Data Availability

Data is available on request.
